# Performance of Multi-City Land Use Regression Models for Nitrogen Dioxide and Fine Particles

**DOI:** 10.1289/ehp.1307271

**Published:** 2014-05-02

**Authors:** Meng Wang, Rob Beelen, Tom Bellander, Matthias Birk, Giulia Cesaroni, Marta Cirach, Josef Cyrys, Kees de Hoogh, Christophe Declercq, Konstantina Dimakopoulou, Marloes Eeftens, Kirsten T. Eriksen, Francesco Forastiere, Claudia Galassi, Georgios Grivas, Joachim Heinrich, Barbara Hoffmann, Alex Ineichen, Michal Korek, Timo Lanki, Sarah Lindley, Lars Modig, Anna Mölter, Per Nafstad, Mark J. Nieuwenhuijsen, Wenche Nystad, David Olsson, Ole Raaschou-Nielsen, Martina Ragettli, Andrea Ranzi, Morgane Stempfelet, Dorothea Sugiri, Ming-Yi Tsai, Orsolya Udvardy, Mihaly J. Varró, Danielle Vienneau, Gudrun Weinmayr, Kathrin Wolf, Tarja Yli-Tuomi, Gerard Hoek, Bert Brunekreef

**Affiliations:** 1 Institute for Risk Assessment Sciences, Utrecht University, Utrecht, the Netherlands; 2 Institute of Environmental Medicine, Karolinska Institutet, Stockholm, Sweden; 3 Institute of Epidemiology I, Helmholtz Zentrum München, German Research Center for Environmental Health, Neuherberg, Germany; 4 Epidemiology Department, Lazio Regional Health Service, Rome, Italy; 5 Center for Research in Environmental Epidemiology (CREAL), Barcelona, Spain; 6 Institute of Epidemiology II, Helmholtz Zentrum München, German Research Center for Environmental Health, Neuherberg, Germany; 7 University of Augsburg, Environmental Science Center, Augsburg, Germany; 8 MRC-PHE Centre for Environment and Health, Department of Epidemiology and Biostatistics, Imperial College London, London, United Kingdom; 9 French Institute for Public Health Surveillance, Saint-Maurice, France; 10 Department of Hygiene, Epidemiology and Medical Statistics, National and Kapodistrian University of Athens, Medical School, Athens, Greece; 11 Danish Cancer Society Research Center, Copenhagen, Denmark; 12 AOU Città della Salute e della Scienza–Center for Cancer Prevention (CPO Piedmont), Turin, Italy; 13 School of Chemical Engineering, National Technical University of Athens, Athens, Greece; 14 IUF Leibniz Research Institute for Environmental Medicine, University of Düsseldorf, Düsseldorf, Germany; 15 Department of Epidemiology and Public Health, Swiss Tropical and Public Health Institute, Basel, Switzerland; 16 Department of Environmental Health, National Institute for Health and Welfare, Kuopio, Finland; 17 School of Environment and Development (Geography), University of Manchester, Manchester, United Kingdom; 18 Department of Public Health and Clinical Medicine, Umeå University, Umeå, Sweden; 19 Centre for Occupational and Environmental Health, University of Manchester, Manchester, United Kingdom; 20 Institute of Health and Society, University of Oslo, Oslo, Norway; 21 Norwegian Institute of Public Health, Oslo, Norway; 22 University of Basel, Basel, Switzerland; 23 Department of Environmental and Occupational Health Sciences, University of Washington, Seattle, USA; 24 Department of Air Hygiene, National Institute of Environmental Health, Budapest, Hungary; 25 Institute of Epidemiology and Medical Biometry, Ulm University, Ulm, Germany; 26 Julius Center for Health Sciences and Primary Care, University Medical Center Utrecht, Utrecht, the Netherlands

## Abstract

Background: Land use regression (LUR) models have been developed mostly to explain intraurban variations in air pollution based on often small local monitoring campaigns. Transferability of LUR models from city to city has been investigated, but little is known about the performance of models based on large numbers of monitoring sites covering a large area.

Objectives: We aimed to develop European and regional LUR models and to examine their transferability to areas not used for model development.

Methods: We evaluated LUR models for nitrogen dioxide (NO_2_) and particulate matter (PM; PM_2.5_, PM_2.5_ absorbance) by combining standardized measurement data from 17 (PM) and 23 (NO_2_) ESCAPE (European Study of Cohorts for Air Pollution Effects) study areas across 14 European countries for PM and NO_2_. Models were evaluated with cross-validation (CV) and hold-out validation (HV). We investigated the transferability of the models by successively excluding each study area from model building.

Results: The European model explained 56% of the concentration variability across all sites for NO_2_, 86% for PM_2.5_, and 70% for PM_2.5_ absorbance. The HV *R*^2^s were only slightly lower than the model *R*^2^ (NO_2_, 54%; PM_2.5_, 80%; PM_2.5_ absorbance, 70%). The European NO_2_, PM_2.5_, and PM_2.5_ absorbance models explained a median of 59%, 48%, and 70% of within-area variability in individual areas. The transferred models predicted a modest-to-large fraction of variability in areas that were excluded from model building (median *R*^2^: NO_2_, 59%; PM_2.5_, 42%; PM_2.5_ absorbance, 67%).

Conclusions: Using a large data set from 23 European study areas, we were able to develop LUR models for NO_2_ and PM metrics that predicted measurements made at independent sites and areas reasonably well. This finding is useful for assessing exposure in health studies conducted in areas where no measurements were conducted.

Citation: Wang M, Beelen R, Bellander T, Birk M, Cesaroni G, Cirach M, Cyrys J, de Hoogh K, Declercq C, Dimakopoulou K, Eeftens M, Eriksen KT, Forastiere F, Galassi C, Grivas G, Heinrich J, Hoffmann B, Ineichen A, Korek M, Lanki T, Lindley S, Modig L, Mölter A, Nafstad P, Nieuwenhuijsen MJ, Nystad W, Olsson D, Raaschou-Nielsen O, Ragettli M, Ranzi A, Stempfelet M, Sugiri D, Tsai MY, Udvardy O, Varró MJ, Vienneau D, Weinmayr G, Wolf K, Yli-Tuomi T, Hoek G, Brunekreef B. 2014. Performance of multi-city land use regression models for nitrogen dioxide and fine particles. Environ Health Perspect 122:843–849; http://dx.doi.org/10.1289/ehp.1307271

## Introduction

Many studies have documented adverse health effects associated with long-term exposure to air pollutants (e.g., Brunekreef and Holgate 2002). With the improvement of the accuracy of geographical data, air pollution models incorporating data from geographical information systems (GIS) are of increasing interest in exposure assessment (Hoek et al. 2008; Jerrett et al. 2005). Land use regression (LUR) modeling is a popular method used for exposure assessment in health studies (Cesaroni et al. 2013; Estarlich et al. 2011; Gehring et al. 2011). LUR modeling is a GIS- and statistics-based method that exploits land use, geographic, and traffic characteristics (e.g., traffic intensity, road length, population density) to explain spatial concentration variations at monitoring sites.

Land use regression models were constructed and used mostly to predict concentrations within metropolitan areas (Hoek et al. 2011; Madsen et al. 2007; Marshall et al. 2008) or small regions (Brauer et al. 2003; Henderson et al. 2007). Often, models have been based on measurements made at a relatively small number of sampling sites (20 to ~ 80 sites). Our recent study showed a positive association between the number of sampling sites and the prediction capability of models for NO_2_ based on 144 sites in the Netherlands (Wang et al. 2012), in agreement with observations for Girona, Spain (Basagaña et al. 2012). At least for some of the reported studies, there is still room to improve the model performances if more sampling sites were selected (Hoek et al. 2008). Several studies have reported the possibilities of building models in large areas in Europe, United States, and Canada (Beelen et al. 2009; Hart et al. 2009; Hystad et al. 2011; Vienneau et al. 2009, 2013). With a large number of sites, these models explained large fractions of NO_2_ variability (61% to ~ 90%) and a modest fraction of the variability of PM (40% to ~ 50%) across all sites. The large-area studies were all based on routine monitoring data. National routine monitoring networks may include only a small number of sites within individual cities. Therefore it may be difficult to evaluate how well a large-area model explains within-city variability. This is relevant for epidemiological studies based in individual cities. A study in Switzerland based on study-specific monitoring suggested that a countrywide model did not perform well within six of the eight geographically diverse study areas (Liu et al. 2012).

The applicability of LUR models can be increased by transferring them to adjacent areas with similar geography and GIS databases where no or few measurements were conducted. The transferability of models has been investigated for local and national models (Allen et al. 2011; Poplawski et al. 2009; Vienneau et al. 2010). Most of the earlier studies recommended using the locally built models, even though transferred models explained variations in concentrations fairly well. This was recommended because all the transferred models were city–city or country–country transfers for which local specific variables were not available, and there was no advantage in the number of sampling sites compared with the locally developed models.

So far, few studies have attempted to explore the performance of LUR models with combined geographical areas in terms of prediction ability and transferability at independent sites and areas—mainly because sufficient, comparable measurement data are lacking. In the context of the European Study of Cohorts for Air Pollution Effects (ESCAPE 2013), we applied a standardized approach for measurements, GIS variable collection, and model development for nitrogen dioxide (NO_2_) and particulate matter (PM) in 36 study areas in Europe (Beelen et al. 2013; Cyrys et al. 2012; Eeftens et al. 2012a, 2012b). We recently published LUR models developed within individual study areas for NO_2_ and PM (Beelen et al. 2013; Eeftens et al. 2012a). The ESCAPE database provides a unique opportunity to address important questions regarding application of LUR models developed for even larger areas. Therefore, the aims of this study are *a*) to develop LUR models for NO_2_, PM_2.5_ (PM with diameter ≤ 2.5 μm), and PM_2.5_ absorbance based on combining the ESCAPE study areas across Europe and across four regions of Europe; *b*) to evaluate the model performances systematically in terms of model fitting and prediction ability; and *c*) to investigate the transferability of the regional and European models to monitoring sites and areas not included in the model building.

## Methods

*Study areas and air pollution measurements.* Details of the ESCAPE study design and the measurement campaign have been described previously (Cyrys et al. 2012; Eeftens et al. 2012b). Briefly, an intensive monitoring campaign was conducted in 36 European study areas between October 2008 and May 2011. ESCAPE included 20 areas with simultaneous measurements of both PM and NO_2_ at 20 sites per area, and at 20 sites where only NO_2_ was measured. In an additional 16 areas, where PM measurements were not available, only NO_2_ measurements were conducted at 40 sites per area. The number of measurement sites was doubled in the large study area of the Netherlands and Belgium. In each area, we chose sampling sites at street, urban background, and regional background locations. Sites were also selected to cover locally important variation—for example, presence of a port or altitude. These sites were selected to represent the spatial distribution of air pollution and residential addresses of participants of cohort studies in these areas. The background sites have been carefully selected to the locations not influenced by local traffic and other local emissions (e.g., industry and port) (Beelen et al. 2013; Eeftens et al. 2012a). Annual average concentrations were calculated from three 2-week samples in the cold, warm, and intermediate seasons. Because the number of samplers was limited, five sites and the references site were measured simultaneously. The measured values were adjusted for temporal trends with data from the continuous reference site in each area by calculating absolute differences between concentrations at monitoring sites and reference sites and using that as adjustment factor (Cyrys et al. 2012; Eeftens et al. 2012b).

**Figure 1:**
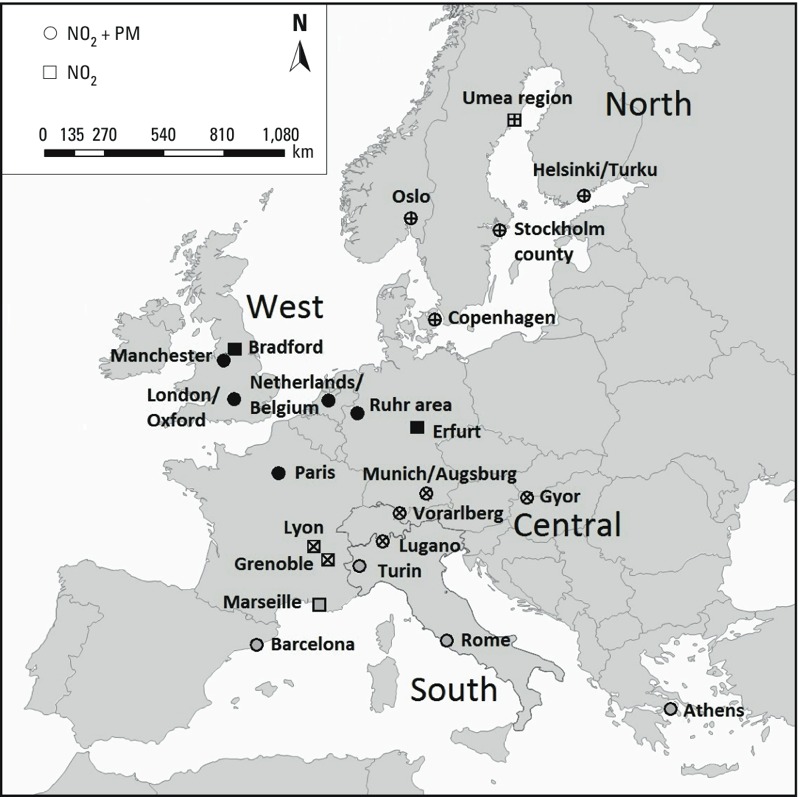
Map of study areas including region ­indication. Symbols: black, West Europe; +, North Europe; ×, Central Europe; open, South Europe

For this paper, we selected the 23 areas (Figure 1) in which traffic intensity variables were available for LUR model building in line with the importance of traffic intensity variables in model development (Beelen et al. 2013). This included 17 of the 20 PM/NO_2_ areas and 6 of the 16 NO_2_-only areas. We allocated the areas to four regions according to the geographic location, the characteristics of the climate, the traffic intensity levels, and the configuration of the cities/country. These regions included five areas in north Europe (Oslo, Norway; Stockholm and Umeå, Sweden; Copenhagen, Denmark; Helsinki/Turku, Finland), seven in the west (Netherlands and Belgium; London, Manchester, and Bradford, UK; Ruhr area and Erfurt, Germany; Paris, France), six in the center (Munich and Vorarlberg, Germany; Györ, Hungary; Lugano, Switzerland; Grenoble and Lyon, France), and five in the south (Turin and Rome, Italy; Athens, Greece; Barcelona, Spain; Marseille, France) (Figure 1, Table 1).

**Table 1:** Study areas.

Code	Type	Region	Study area
NOS	PM/NO_2_	North	Oslo, Norway
SST	PM/NO_2_	North	Stockholm, Sweden
FIH	PM/NO_2_	North	Helsinki/Turku, Finland
DCO	PM/NO_2_	North	Copenhagen, Denmark
SUM	NO_2_	North	Umeå, Sweden
UKM	PM/NO_2_	West	Manchester, UK
UKO	PM/NO_2_	West	London, Oxford, UK
BNL	PM/NO_2_	West	Netherlands and Belgium
GRU	PM/NO_2_	West	Ruhr area, Germany
GRE	NO_2_	West	Erfurt, Germany
UKB	NO_2_	West	Bradford, UK
FPA	PM/NO_2_	West	Paris, France
GMU	PM/NO_2_	Central	Munich, Germany
AUV	PM/NO_2_	Central	Vorarlberg, Austria
FLY	NO_2_	Central	Lyon, France
HUG	PM/NO_2_	Central	Györ, Hungary
SWL	PM/NO_2_	Central	Lugano, Switzerland
FGR	NO_2_	Central	Grenoble, France
ITU	PM/NO_2_	South	Turin, Italy
IRO	PM/NO_2_	South	Rome, Italy
SPB	PM/NO_2_	South	Barcelona, Spain
FMA	NO_2_	South	Marseille, France
GRA	PM/NO_2_	South	Athens, Greece

For this study we selected NO_2_ and PM_2.5_ absorbance to represent traffic-related air pollution, and PM_2.5_ for a more complex mixture of sources. NO_2_ was measured using Ogawa badges following the Ogawa analysis protocol (V 3.98; Ogawa & Co., Pompano Beach, FL USA). PM_2.5_ samples were collected on preweighted filters using Harvard impactors, and were then used to measure absorbance (Cyrys et al. 2012; Eeftens et al. 2012b).

*Predictor variables.* We extracted values for the GIS predictor variables at the locations of sampling sites using ArcGIS (ESRI, Redlands, CA, USA). Details of the predictor variables have been described in previous papers (Beelen et al. 2013; Eeftens et al. 2012a). Briefly, the predictor variables were derived from both centrally available Europe-wide GIS databases and GIS data collected by the local centers using standard definitions.

Central GIS predictor variables included road network, land use, population density, and altitude data. The digital road network was obtained from EuroStreets version 3.1 (EuroStreets 2013) for the year 2008. The total lengths of all roads and major roads were calculated within a buffer size of 25, 50, 100, 300, 500, or 1,000 m. Traffic intensity data were not available for this road network. Land use variables were derived from the European Corine Land Cover (European Environment Agency 2000) database for the year 2000 for the buffer sizes of 100, 300, 500, 1,000 and 5,000 m. Digital elevation data were obtained through the Shuttle Radar Topographic Mission (SRTM) (CGIAR Consortium for Spatial Information 2013). Detailed road network with linked traffic intensity for all road links were obtained from local sources for all 23 areas. Local land use, population density, altitude, and other local variables were also locally extracted for modeling.

For the regional and European models, we pooled the data by including all the central GIS predictors and the local traffic variables with traffic intensity. We combined the centrally available land use variables high and low residence density, and the natural and urban green variables because not all the areas contained them separately. We made efforts to incorporate more local common variables for specific regions to capture regional variations. We included regional background concentrations of NO_2_, PM absorbance, and PM_2.5_ as the mean of the measured concentrations at ESCAPE regional background sites (1–20) in each local study area to characterize the spatial differences between study areas. In the Netherlands, regional background concentrations were interpolated from regional background sites throughout the country because background concentrations may vary at such a large scale. In total, 49 variables were evaluated at the European level and 54, 53, 54, and 64 variables in the north, west, middle, and south regions, respectively (see Supplemental Material, Table S1).

*Model development.* A total of 960 NO_2_ sites and 356 PM sites (four sites were missing due to failed campaign) were available for modeling from 23 and 17 study areas, respectively. Detailed procedures of the NO_2_ and PM model development have been published elsewhere (Beelen et al. 2013; Eeftens et al. 2012a). The regional and European models were developed using the same strictly standardized approaches. Briefly, a supervised stepwise regression was used to develop the LUR model. We first evaluated univariate regression of the annual concentrations by entering all potential predictor variables. We forced the regional background concentration variable in the first step (for the European and regional models). Then the variable that produced the highest adjusted *R*^2^ and which had the *a priori*–defined direction of effect (e.g., positive for traffic intensity) was selected as the second predictor. Second, the remaining variables were added separately, and we assessed whether the variable with the highest increase in adjusted *R*^2^ improved the model by at least 1%. This process continued until no more variables with the *a priori*–specified sign could increase the model-adjusted *R*^2^ by at least 1%. In the final step, we excluded variables that had a *p*-value > 0.1. We checked whether the variance inflation factor was < 3 to avoid multicollinearity.

*Model evaluations.* We used three approaches for model evaluation:

We investigated the model fit at individual study areas by applying the European/regional model to the sites of each area that were used for modeling. The Model_intra_ *R*^2^ shows the within-area variations explained by the European/regional models, which are directly comparable with the *R*^2^ of city-specific models. The Model_intra_ *R*^2^ is important for studies conducted within individual cities that use the European/regional model. The overall *R*^2^ is relevant for multi-city studies that exploit both within- and between-city variability of air pollution contrasts. The Model_intra_ *R*^2^ is important for European studies such as ESCAPE because cohorts were located within a city or small area, and cohort-specific epidemiological analyses were conducted.
Cross-validation (CV) is an internal validation for testing the stability of model fit. We conducted leave-one-area-out-cross-validation (LOAOCV) by leaving out all observations from a complete area of *n* study areas (*n* = 23 for NO_2_ and 17 for PM), refitting the model based on the remaining M-1 areas, and investigating the agreement between predicted and observed concentrations for each area that was left out. This was iterated M times, and the LOAOCV reflects the heterogeneity of model fit due to regional variations between study areas. We do not report LOAOCV that was almost identical to the model *R*^2^ probably because of the large training data set.
The hold-out validation (HV) is an evaluation of model predictive power to independent sites not used for model building. In contrast with CV, HV reflects the prediction ability of models to the cohort addresses within the areas on which the models had been established. As a test, we divided the full set into two parts; the training sets were used for modeling and the remaining test sets were used for external evaluation. For NO_2_, we developed models using the PM/NO_2_ sites with 20–40 sites per area (480 sites in total) as training sets and the remaining 480 NO_2_-only sites as test sets. For PM_2.5_ and PM_2.5_ absorbance, a randomly selected 25% of the PM sites stratified by study area were used for validation purpose because we had fewer sites available for PM model building than for NO_2_ model building. The HV *R*^2^ is the squared Pearson correlation between predictions and observations at the independent sites throughout the whole study area. We calculated the HV *R*^2^ by truncating the values of predictors in the test data sets that were outside the range of the values observed in the data set for model development, to prevent unrealistic predictions based on model extrapolations (Wang et al. 2012). Prediction errors were estimated by root mean squared error (RMSE). In our previous study, the same NO_2_ training and test sets were used for the ESCAPE city-specific model evaluations individually in each study area (Wang et al. 2013). Therefore, a fair comparison of prediction ability (HV *R*^2^) between the European model and the city-specific models can be conducted using the same test sets for HV. The comparison was available only for NO_2_ due to relatively large number of sampling sites.

*Transferability of LUR models.* To evaluate the prediction abilities of the regional/European models to independent individual study areas, we developed the regional and European models by excluding one area at a time and applied the transferred models directly to the sites of the area that was left out. Therefore, 23 NO_2_ models and 17 PM models were built until each of the study areas had been excluded once from model building.

The TRANS_intra_ *R*^2^ is the squared Pearson correlation between observed and predicted values in each of the remaining areas that was excluded from modeling. The TRANS_intra_ *R*^2^ is different from the Model_intra_ and the LOAOCV *R*^2^ because the measurements conducted in the respective validation areas were completely left out from model development.

## Results

*NO_2_ and PM concentrations.* Table 2 shows the concentration distributions of NO_2_ and PM metrics across the study areas by site types. Substantial spatial variations were found for all the pollutants across Europe. The variability was larger for NO_2_ than for PM_2.5_. The spatial variability for PM_2.5_ absorbance was intermediate between PM_2.5_ and NO_2_. Concentration contrasts were larger at the street sites for NO_2_ and PM_2.5_ absorbance than at the urban and regional background sites. Concentration contrasts for PM_2.5_ were more similar at all the site types, suggesting an influence of multiple sources in addition to traffic.

**Table 2:** Distributions of measured annual average NO_2_ and PM concentrations across Europe.

Pollutant and site type	*n*^*a*^	Minimum	25th	Median	75th	Maximum
NO_2_ (μg/m^3^)
Street sites	454	11.80	25.48	33.98	49.90	109.00
Urban background	414	3.03	15.38	22.88	30.67	57.63
Regional background	92	1.53	9.56	15.48	17.98	32.87
PM_2.5_ (μg/m^3^)
Street sites	166	7.87	12.03	17.18	21.17	36.30
Urban background	144	5.62	10.97	15.87	18.62	32.59
Regional background	47	4.42	11.20	13.86	16.64	23.24
PM_2.5_ absorbance (10^–5^/m)
Street sites	166	0.78	1.63	2.16	2.81	5.09
Urban background	144	0.53	1.23	1.67	2.01	3.03
Regional background	47	0.33	0.92	1.16	1.45	2.37
25th and 75th are percentiles. ^***a***^Total number of sites in the study areas.						

*Models in combined areas.* Table 2 shows the model details of NO_2_, PM, and PM_2.5_ absorbance combining all the European study areas. The NO_2_, PM_2.5_, and PM_2.5_ absorbance models explained 56%, 86%, and 70%, respectively, of the variation across all sites, which includes both within and between area variations (overall model *R*^2^). The LOAOCV *R*^2^ was 5% and 6% lower than the model *R*^2^ for NO_2_ and PM_2.5_, respectively, and was identical to the model *R*^2^ for PM_2.5_ absorbance. The HV *R*^2^s (50% training vs. 50% test sites for NO_2_, 75% training vs. 25% test sites for PM metrics) were slightly smaller than or nearly identical to the model *R*^2^s, explaining 54%, 80%, and 70% for NO_2_, PM_2.5_, and PM_2.5_ absorbance at the independent validation sites respectively (see Supplemental Material, Table S2). The HV *R*^2^ did not change if the predictor range was not truncated because only one site for NO_2_ model was truncated. The HV RMSE values were close to the values of LOAOCV RMSE for NO_2_ and PM metrics. The RMSE values were relatively small compared with the range of measurements as shown in Supplemental Material, Table S2. The median HV *R*^2^ of the European NO_2_ model at individual study areas was identical to those of the city-specific models reported by Wang et al. (2013) (see Supplemental Material, Figure S1). In the Turin and Paris areas with a low hold-out evaluation *R*^2^, for example, the HV *R*^2^s of the European model were considerably larger than those of the city-specific models.

All the models in Table 3 included traffic intensity variables. The regional background concentration explained a large fraction (71%) of variation in PM_2.5_ documenting the importance of between-area differences for PM_2.5_ compared with that for the more traffic-related pollutants NO_2_ and PM_2.5_ absorbance.

**Table 3:** European models for NO_2_, PM_2.5_, and PM_2.5_ absorbance.

Predictors	Partial *R*^2^	β^*a*^	Model_intra_^*b*^ *R*^2^/IQR	LOAOCV *R*^2^/RMSE
NO_2_ (*n*^*c*^ = 960, final model *R*^2^ = 0.56)			0.59/0.19	0.50/8.49 (μg/m^3^)
Regional background concentration	0.08	2.63E-01
Traffic load in 50 m	0.35	2.44E-06
Road length in 1,000 m	0.50	2.74E-04
Natural and green in 5,000 m	0.55	–2.84E-07
Traffic intensity on the nearest road	0.56	2.21E-04
Intercept		1.38E+01
PM_2.5_ (*n*^*c*^ = 356, final model *R*^2^ = 0.86)			0.48/0.16	0.81/2.38 (μg/m^3^)
Regional background concentration	0.71	9.73E-01
Traffic load between 50 m and 1,000 m	0.81	4.75E-09
Traffic load in 50 m	0.84	5.28E-07
Road length in 100 m	0.86	2.12E-03
Intercept		3.06E-01
PM_2.5_ absorbance (*n*^*c*^ = 356, final model *R*^2^ = 0.70)			0.70/0.19	0.70/0.45 (10^–5/^m)
Regional background concentration	0.28	9.06E-01
Traffic load in 50 m	0.58	2.07E-07
Road length in 500 m	0.67	2.90E-05
Natural and green in 5,000 m	0.69	–9.63E-09
Traffic load between 50 m and 1,000 m	0.70	4.20E-10
Intercept		2.95E-01
^***a***^Coefficients of predictor variables in the models. ^***b***^The Model_intra_ *R*^2^s show the median and interquartile range (IQR) of the within-area variability explained by the European model in individual areas. ^***c***^Number of monitored sites available for model building.				

The regional models performed equally well as the European models in all regions except Southern Europe, where none of the models performed well in terms of the predictions to the independent sites (HV *R*^2^: 0–0.23) (see Supplemental Material, Table S3). Reassigning Turin from south Europe to the central Europe region only slightly changed the results.

**Figure 2:**
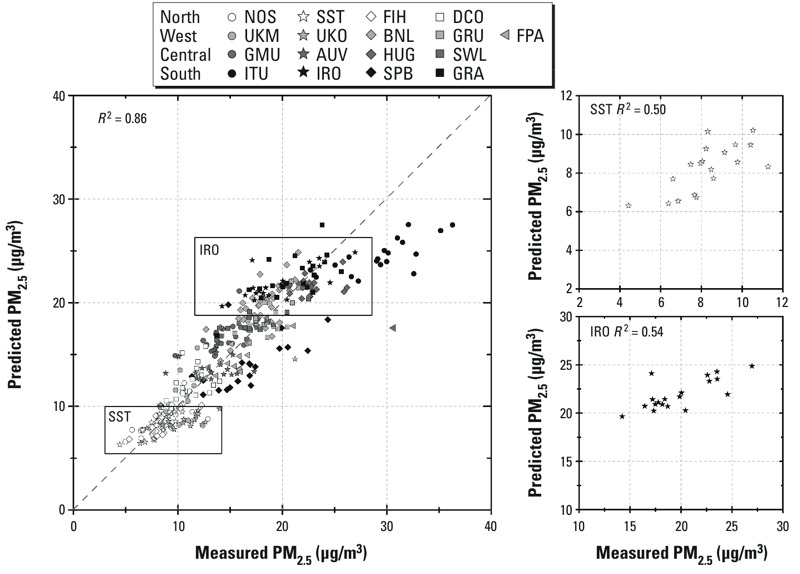
Scatter plots of predicted and measured PM_2.5_ with study areas color and symbol coded and two city-specific examples, Stockholm (SST) and Rome (IRO). See Table 1 for study area codes.

As shown in Table 3, the median within-area variability (Model_intra_ *R*^2^) explained by the European model for NO_2_ and PM_2.5_ absorbance at individual study areas was similar to the overall model *R*^2^, suggesting predominant sources of local emissions. For PM_2.5_, the median Model_intra_ *R*^2^ was much lower than the overall model *R*^2^ (0.48 vs. 0.86). Figure 2 (see also Supplemental Material, Figure S2) presents the correlation between predicted and measured PM_2.5_, PM_2.5_ absorbance, and NO_2_ by study areas. As the figures show, the variation of PM_2.5_ between areas was substantial compared to the within areas variation (e.g., low PM_2.5_ values in northern European cities such as Stockholm and high PM_2.5_ values in southern European cities such as Rome). On the contrary, for NO_2_ and PM_2.5_ absorbance, variation within areas was substantial compared with the variation between areas (see Supplemental Material, Figure S2). The observations are more underpredicted within individual areas for PM metrics (median regression slope: PM_2.5_, 0.47; PM_2.5_ absorbance, 0.57; NO_2_, 0.56) than across the whole European study areas (regression slope: PM_2.5_, 0.85; PM_2.5_ absorbance, 0.70; NO_2_, 0.57).

**Figure 3:**
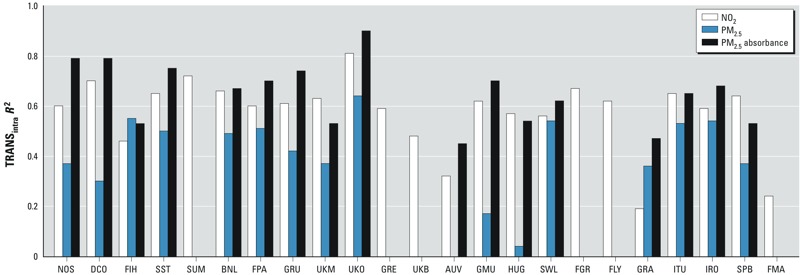
Transferability (TRANS_intra_ *R*^2^) of the European models for NO_2_ and PM in the 23 study areas. See Table 1 for study area codes.

*Transferability.* Table 4 shows the performance of the models that used all monitoring data excluding one area at the time. These models explained on average 57%, 84%, and 69% variability of NO_2_, PM_2.5_, and PM_2.5_ absorbance respectively. The model structures and *R*^2^s were similar to the models in Table 3, which were based on all study areas. They included the same variable categories but with, to some extent, different buffer sizes. The models predicted the spatial variations of NO_2_ and PM_2.5_ absorbance well in the areas not used for model building, with median TRANS_intra_ *R*^2^s of 0.59 for NO_2_ and 0.67 for PM_2.5_ absorbance. Transferability was less for PM_2.5_ with a median *R*^2^ of 0.42. The same pattern was found for the model *R*^2^ focusing on within-area variability only (Model_intra_). The variation in prediction *R*^2^s was relatively small for NO_2_, with an interquartile range (IQR) of 0.09, but larger for PM_2.5_ (IQR, 0.17) and PM_2.5_ absorbance (IQR, 0.21), showing that predictions were less comparable for the two PM metrics. The variation is shown in Figure 3 (see also Supplemental Material, Figure S3). Interestingly, this did not depend so much on area as on the specific combination of area and component. For example, the areas in Hungary (GyÖr), Germany (Munich), and Austria (Vorarlberg) showed decent model fit and predictability for NO_2_ and PM_2.5_ absorbance, but almost no model fit and predictability for PM_2.5_. The transferred regional models showed similar characteristics as those of the European models, whereas the median TRANS_intra_ *R*^2^ was slightly lower (see Supplemental Material, Table S4).

**Table 4:** Transferability of European models to areas that were not used for model building [median (IQR)]

Pollutant	Model *R*^2^	Model_intra_^*a*^ *R*^2^	TRANS_intra_^*b*^	
			*R*^2^	RMSE
NO_2_	0.57 (0.01)	0.59 (0.19)	0.59 (0.09)	5.58 (2.28)
PM_2.5_	0.84 (0.01)	0.48 (0.16)	0.42 (0.17)	1.14 (0.58)
PM_2.5_ absorbance	0.69 (0.01)	0.70 (0.19)	0.67 (0.21)	0.23 (0.07)
IQR, interquartile range. ^***a***^Model_intra_ *R*^2^: *R*^2^ of within-area variation explained by European model, with the same data as in Table 2. ^***b***^TRANS_intra_: squared correlations and RMSE between the predictions and observations at independent areas.				

## Discussion

In this study we developed LUR models for NO_2_, PM_2.5_, and PM_2.5_ absorbance, with combined measurement data from 23 study areas across Europe. For NO_2_ and PM_2.5_ absorbance, these models predicted spatial variations in areas not commonly used for model building. For PM_2.5_, prediction *R*^2^s were moderate for intraurban variation, though in some areas in central Europe prediction *R*^2^s were low. The overall *R*^2^ including both between- and within-study area variability was high for PM_2.5_ and PM_2.5_ absorbance and more moderate for NO_2_.

*Comparisons with other large area studies.* Our European models performed comparable or even better in predictions of NO_2_ and PM_2.5_ than other large area studies (see Supplemental Material, Table S5) (Beckerman et al. 2013; Beelen et al. 2009; Bergen et al. 2013; Hystad et al. 2011; Novotny et al. 2011; Sampson et al. 2013; Vienneau et al. 2013). For PM_2.5_ absorbance, this is the first report of LUR models in such a large geographical area. Model *R*^2^s are difficult to compare because studies differed in study area, model development strategies, scale of prediction, offered predictor variables, and number of sites. Consistent across studies, the regional background predicted a small fraction of variability for NO_2_ and a large fraction for PM_2.5_. For intracity model *R*^2^, our NO_2_ European model exhibited performance (Model_intra_ *R*^2^ = 0.59) comparable with that of the Canadian national model in seven specific areas (Edmonton, Montreal, Sarnia, Toronto, Victoria, Vancouver, and Winnipeg), with Model_intra_ *R*^2^ of 0.43 (Hystad et al. 2011). We observed no heterogeneity of model fit across study areas in the European model (LOAOCV *R*^2^s were close to the model *R*^2^).

Our European and regional models have several strengths compared with previous European models that modeled concentration in 1 × 1 km grids (Beelen et al. 2009; Vienneau et al. 2009) or more recently 100 × 100 m (Vienneau et al. 2013): *a*) We modeled small-scale variation using sampling sites that were selected according to a standard method to cover intraurban concentration contrasts. *b*) We included multiple pollutants (PM_2.5_, PM absorbance), which were much less available or measured with different methods from routine monitoring networks in Europe. *c*) We incorporated local traffic intensity data not available in Europe-wide databases (land use and road length data only). All the models included traffic intensity variables, improving prediction ability (HV *R*^2^) over models not having local traffic intensity data (but potentially road length)—for example, from 0.46 to 0.54 for NO_2_.

The poor performance of the south European model may be attributed to the large heterogeneity of model fit (low LOAOCV *R*^2^) across south European study areas in which the concentrations in Athens were overestimated more than those of the other study areas. More formal methods, such as hierarchical cluster analysis to define regions, could be explored to improve comparability of regions.

Our PM_2.5_ European model explained a median of 48% within-area variations compared with the overall model *R*^2^ of 86%, which was largely explained by substantial differences in regional background concentrations. This was consistent with the *R*^2^s of the Canadian and American PM_2.5_ model (46% and 63%), of which the satellite data alone explained 41% and 52% of the variability, respectively (Beckerman et al. 2013; Hystad et al. 2011) (see Supplemental Material, Table S5). PM_2.5_ is well known to be a regional pollutant with a large fraction of secondary aerosol, not explained well by the local GIS and traffic variables typically available for LUR model building. This suggested that for pollutants (e.g., PM_2.5_) with much larger overall than within-city *R*^2^, joint analyses of cohorts including between-city exposure components might be advisable. This does require the assumption of sufficient comparability of cohorts across Europe. Other methods such as partial least squares regression may help to increase the prediction ability of models (Sampson et al. 2013).

*Comparison with ESCAPE city-specific models.* NO_2_ and PM models based on small training sets and a large number of predictor variables overestimate predictive ability in independent test sets, though still explaining fairly large fractions (50% to ~ 60%) of spatial variability (Wang et al. 2012, 2013). HV *R*^2^s of the European models developed on a large number of sites were very similar to the model *R*^2^. The average differences of the model *R*^2^s and HV *R*^2^s were just 2%, 6%, and 0% for NO_2_, PM_2.5_, and PM_2.5_ absorbance. The slightly larger drop for PM_2.5_ could be attributable to more sources affecting PM_2.5_ compared with NO_2_ and PM_2.5_ absorbance.

The ESCAPE city-specific models that have been published previously using local specific variables explained a median of 82%, 71%, and 89% of the concentration variations for NO_2_, PM_2.5_, and PM_2.5_ absorbance (Beelen et al. 2013; Eeftens et al. 2012a). This is higher than the *R*^2^ of within-area variability explained by the European models in Table 2 (Model_intra_ *R*^2^: 59%, 48%, 70% respectively). The average differences between the individual city-specific model *R*^2^ (Beelen et al. 2013; Eeftens et al. 2012a) and the intraurban *R*^2^ (see Supplemental Material, Figure S3) are 24%, 24%, and 17% for NO_2_, PM_2.5_, and PM_2.5_ absorbance respectively. Because model *R*^2^ overestimates predictive ability, especially when developed for a relatively small number of sites (Wang et al. 2012, 2013), the comparison between local and European models should not be based on model *R*^2^ but HV *R*^2^ at independent sites. Comparison of the prediction ability between the European and city-specific models is feasible only for NO_2_, which suggested that the European and city-specific model had similar median prediction ability to the external sites not used for modeling. The HV *R*^2^ in some cities [e.g., Turin (ITU) and Paris (FPA)] that had poor predictions by the city-specific model may be improved substantially by the European model. We cannot draw a firm conclusion about one or the other approach being more reliable because comparisons for PM models were infeasible. The European model may reduce bias in health estimates because of relatively large number of sampling sites and small number of variables compared with the city-specific models (Basagaña et al. 2013).

Most of the combined models included traffic variables in both large (≥ 500 m) and small buffers (≤ 50 m), representing general area characteristics as well as localized influences. In contrast to the study-area specific ESCAPE models (Beelen et al. 2013; Eeftens et al. 2012a), none of our European models included population/residence density, but instead selected road length in large buffers, which likely also represents urban–rural difference in terms of population distributions (de Hoogh et al. 2013). In our GIS data set, the squared correlation *R*^2^ between road length and population density is 0.46 within a 1,000-m buffer but is only 0.13 within a 100-m buffer. Road length variables in large buffers therefore represent various aspects of “total human activity” such as traffic, heating, population density.

*Transferability of combined models.* Previous studies on the transferability of LUR models were mainly focusing on city-to-city or country-to-country transferability. Briggs et al. (2000) concluded that the SAVIAH (Small-Area Variations In Air Quality and Health) models could be applied to other UK cities after calibrating with data from a few monitoring sites. Poplawski et al. (2009) and Allen et al. (2011) observed that local calibration may improve the predictions of the Canadian city-specific models to a few other comparable cities in Canada and the United States. Vienneau et al. (2010) found reasonable transferability of British and the Dutch models between these two countries. All the previous studies concluded that the performances of the transferred models were worse than those of the local source models.

Our results show prediction capabilities for the traffic-related pollutants NO_2_ and PM_2.5_ absorbance that are on par with those documented, in terms of HV *R*^2^s, with previous local exercises (Basagaña et al. 2012; Wang et al. 2012). This might be attributable to the fact that the ESCAPE study used highly standardized monitoring and GIS data for measurement, data collection, and model building across all areas. This suggests that our combined models can be carefully applied to other areas in Europe with common predictors, similar geographies, and availability of consistent regional background concentration within the region. Because the locations are well characterized, any candidate background location in a new area can be judged against the same criteria. Obviously, this will only work when the pollution characteristics or components are actually measured in the new area. In practice, this means that modeling of new areas will in most cases be restricted to NO_2_/nitrogen oxides and PM_10_ (PM ≤ 10 μm) and, in fewer areas (in Europe), to PM_2.5_ and PM absorbance. Satellite data have large spatial coverage and have improved NO_2_ and PM_10_ European models based upon routine monitor data by 5% and 11% (Vienneau et al. 2013). Satellite data could be used in the future to estimate background concentrations in new locations.

In some individual areas of central Europe, the European model performed poorly for PM_2.5_, however, probably due to lack of an important local predictor variable (e.g., residential density in Munich and Vorarlberg, industry in Hungary, or altitude in Vorarlberg). Therefore, caution is needed when transferring the European models to cities for which the European model lacks predictor variables that are known to be important sources of variation locally. The poor performance in a few areas suggests that the value of the European model is especially in multicenter analyses such as ESCAPE compared with studies of individual areas.

*Applications in epidemiological studies.* The overall *R*^2^ of the European model was highest for PM_2.5_ and lowest for NO_2_. In contrast, for within-city variation, the model had the lowest predictive ability for PM_2.5_, though it was still fairly high (median *R*^2^ = 0.48). The PM_2.5_ absorbance model explained both large fractions of variability overall and within-city. The high overall *R*^2^ suggests that the model can be used in pooled analyses of health data, exploiting exposure contrasts between study areas. Using between-city comparisons would be especially useful to increase PM_2.5_ contrasts. For ESCAPE, where the health findings based on these local exposure models are currently being published (Beelen et al. 2014; Raaschou-Nielsen et al. 2013), the model offers the possibility for pooled analyses. Pooled analyses have not been conducted so far, partly because of concerns of comparability of the diverse cohorts across Europe. There is also the possibility to include new study populations from areas where local measurements were never conducted but relevant predictor variables are available. For exposure assessment with LUR models, efforts are mainly in the sampling campaign and GIS data collection.

## Conclusions

European LUR models for NO_2_, PM_2.5_, and PM_2.5_ absorbance were found to have reasonable power to predict spatial variations of these components in areas not used for model building.

## Supplemental Material

(4.4 MB) PDF
