# Erratum: “Performance of Multi-City Land Use Regression Models for Nitrogen Dioxide and Fine Particles”

**DOI:** 10.1289/ehp.122-A322

**Published:** 2014-12-01

Wang et al. discovered an error in their article “Performance of Multi-City Land Use Regression Models for Nitrogen Dioxide and Fine Particles” [Environ Health Perspect 122:843–849 (2014); http://dx.doi.org/10.1289/ehp.1307271]. In Table 3, beta values for the NO_2_ model were incorrect. The corrected table appears below.

**Table 3:** European models for NO_2_, PM_2.5_, and PM_2.5_ absorbance.

Predictors	Partial *R*^2^	β^*a*^	Model_intra_^*b*^ *R*^2^/IQR	LOAOCV*R*^2^/RMSE
NO_2_ (*n*^*c*^ = 960, final model *R*^2^ = 0.56)			0.59/0.19	0.50/8.49 (μg/m^3^)
Regional background concentration	0.08	2.63E-01
Traffic load in 50 m	0.35	2.44E-06
Road length in 1,000 m	0.50	2.74E-04
Natural and green in 5,000 m	0.55	–2.84E-07
Traffic intensity on the nearest road	0.56	2.21E-04
Intercept		1.38E+01
PM_2.5_ (*n*^*c*^ = 356, final model *R*^2^ = 0.86)			0.48/0.16	0.81/2.38 (μg/m^3^)
Regional background concentration	0.71	9.73E-01
Traffic load between 50 m and 1,000 m	0.81	4.75E-09
Traffic load in 50 m	0.84	5.28E-07
Road length in 100 m	0.86	2.12E-03
Intercept		3.06E-01
PM_2.5_ absorbance (*n*^*c*^ = 356, final model *R*^2^ = 0.70)			0.70/0.19	0.70/0.45 (10^–5^/m)
Regional background concentration	0.28	9.06E-01
Traffic load in 50 m	0.58	2.07E-07
Road length in 500 m	0.67	2.90E-05
Natural and green in 5,000 m	0.69	–9.63E-09
Traffic load between 50 m and 1,000 m	0.70	4.20E-10
Intercept		2.95E-01
^***a***^Coefficients of predictor variables in the models. ^***b***^The Model_intra_ *R*^2^s show the median and interquartile range (IQR) of the within-area variability explained by the European model in individual areas. ^***c***^Number of monitored sites available for model building.				

The authors regret the error.

