# Supplementary material for: Performance of Multi-City Land Use Regression Models for Nitrogen Dioxide and Fine Particles
Source: Environ Health Perspect. 2014 May 2;122(8):843–9. doi: 10.1289/ehp.1307271 (PMC4123024; doi:10.1289/ehp.1307271)
Supplement: (4.4 MB) PDF [file ehp.1307271.s001.pdf]

Supplemental Material

Performance of Multi-City Land Use Regression Models for Nitrogen Dioxide and Fine Particles

Meng Wang, Rob Beelen, Tom Bellander, Matthias Birk, Giulia Cesaroni, Marta Cirach, Josef Cyrys, Kees de Hoogh, Christophe Declercq, Konstantina Dimakopoulou,10 Marloes Eeftens, Kirsten T. Eriksen, Francesco Forastiere, Claudia Galassi, Georgios Grivas, Joachim Heinrich, Barbara Hoffmann, Alex Ineichen, Michal Korek, Timo Lanki, Sarah Lindley, Lars Modig, Anna Mölter, Per Nafstad, Mark J. Nieuwenhuijsen, Wenche Nystad, David Olsson, Ole Raaschou-Nielsen, Martina Ragettli, Andrea Ranzi, Morgane Stempfelet, Dorothea Sugiri, Ming-Yi Tsai, Orsolya Udvardy, Mihaly J. Varró, Danielle Vienneau, Gudrun Weinmayr, Kathrin Wolf, Tarja Yli-Tuomi, Gerard Hoek, and Bert Brunekreef

Table of Contents **Page**

Table S1. List of predictor variables for model development, buffer sizes and a priori defined direction of effect 3

Table S2. Descriptive of European model performances for NO_2 and PM metrics using 50% NO_2 training sets and 75% PM training sets for modeling and the remaining 50% and 25% test sets for hold-out validation 4

Table S3. Descriptive of model performances at regional scales using full number of sites 5

Table S4. Transferability of the regional models to the independent areas not used for model building [Median(IQR)] 8

Table S5. Model performances of previous large-scale LUR models 9

Figure S1. Comparison of HV R^2 between the European model and the ESCAPE city-specific models for NO_2 in 23 study areas as well as median and inter quartile range **10**

Figure S2. Scatterplot of predicted and measured of NO_2 and $PM_{2.5}$ absorbance with study areas color and symbol coded **11**

Figure S3. $Model_{intra}$ R^2 of the European models for NO_2 and PM in the 23 study areas **12**

References **13**

Table S1. List of predictor variables for model development, buffer sizes and a priori defined direction of effect.

Region[a]	Variable	Buffer size (m)	Direction
All	High and low residential density	100, 300, 500, 1000, 5000	+
All	Port	300, 500, 1000, 5000	+
All	Industry	300, 500, 1000, 5000	+
All	Urban green and natural areas	100, 300, 500, 1000, 5000	-
All	Squared root of altitude	-	-
All	Road length	50, 100, 300, 500, 1000	+
All	Major road length	50, 100, 300, 500, 1000	
All	Traffic intensity in the nearest road	NA	+
All	(Squared) Inverse distance to the nearest road	NA	+
All	(Squared) Invest distance to the nearest road*traffic intensity in the nearest road	NA	+
All	Traffic intensity in the major road	NA	+
All	(Squared) Inverse distance to the nearest major road	NA	+
All	(Squared) Invest distance to the major road *traffic intensity in the major road	NA	+
All	Total traffic load of roads in a buffer [sum of (traffic intensity * length of all segments)]	50, 100, 300, 500, 1000	+
All	Total traffic load of major roads in a buffer [sum of (traffic intensity * length of all segments)]	50, 100, 300, 500, 1000	+
NE,WE,SE	Population	100, 300, 500, 1000, 5000	+
CE,SE	Urban green	100, 300, 500, 1000, 5000	-
CE,SE	Natural areas	100, 300, 500, 1000, 5000	-
SE	High residential density	100, 300, 500, 1000, 5000	+
SE	Low residential density	100, 300, 500, 1000, 5000	+

[a]All: all study areas; NE: north Europe; WE: west Europe; CE: central Europe; SE: south Europe.

Table S2. Descriptive of European model performances for NO_2 and PM metrics using 50% NO_2 training sets and 75% PM training sets for modeling and the remaining 50% and 25% test sets for hold-out validation.

Model and determinants	Partial R^2	Beta	HV[a] R^2/RMSE
**NO_2 (μg/m^3) (n = 480[b])**			0.54/11.20
Regional background concentration	0.08	3.36E-01	
Traffic load in 50m	0.37	2.60E-06	
Road length in 1000m	0.52	2.65E-04	
Natural and green in 5000m	0.55	-2.19E-07	
Traffic intensity on the nearest road	0.57	1.90E-04	
Intercept		1.10E+01	
**$PM_{2.5}$ (μg/m^3) (n = 270[b])**			0.80/2.78 (μg/m^3)
Regional background concentration	0.71	9.63E-01	
Traffic load between 50m and 1000m	0.82	5.37E-09	
Road length in 50m	0.84	6.89E-03	
Traffic load in 50m	0.86	4.94E-07	
Intercept		4.72E-01	
**$PM_{2.5}$ Absorbance ($10^{-5}m^{-1}$) (n = 270[b])**			0.70/0.45 ($10^{-5}m^{-1}$)
Regional background concentration	0.29	9.58E-01	
Traffic load in 50m	0.56	2.13E-07	
Road length in 500m	0.66	3.53E-05	
Industry in 5000m	0.68	2.50E-08	
Natural and green in 5000m	0.69	-8.65E-09	
Intercept		1.11E-01	

[a]The HV R^2s represent the correlation between predicted and measured concentrations at validation monitoring sites not used for model building (50% for NO_2, 25% for PM metrics, see methods section). [b]N: number of training sites for modeling.

Table S3. Descriptive of model performances at regional scales using full number of sites.

Region[a]/determinants	Partial R^2	Beta	$Model_{intra}$[b] R^2/IQR	LAOCV R^2	HV[c] R^2
**NE**					
NO_2 (N^d=200, final model R^2=0.61)			0.63/0.15	0.52	0.57
Regional background concentration	0.20	9.75E-01			
Traffic load between 50 and 300m	0.48	8.45E-08			
Traffic load in 50m	0.55	2.64E-06			
Road length in 1000m	0.60	1.19E-04			
Traffic load in 300 and 1000m	0.61	2.06E-08			
Intercept		2.34E-01			
$PM_{2.5}$ (N^d=78, final model R^2=0.70)					
Regional background concentration	0.28	5.39E-01	0.68/0.25	0.59	0.60
Natural and green in 1000m	0.64	-1.03E-06			
Traffic density*inverse distance to the nearest road	0.67	2.04E-04			
Road length between 50 and 500m	0.69	1.28E-04			
Major road length in 50m	0.70	9.17E-03			
Intercept		4.26E+00			
$PM_{2.5}$ absorbance (N^d=78, final model R^2=0.69)			0.80/0.11	0.02	0.69
Regional background concentration	0.12	6.77E-01			
Traffic load in 50m	0.50	1.12E-07			
Road length in 500m	0.59	2.26E-05			
Natural and green in 5000m	0.64	-1.00E-08			
Inverse distance to major road	0.69	1.49E+00			
Intercept		5.57E-01			
**WE**					
NO_2 (N^d=320, final model R^2=0.64)			0.65/0.29	0.54	0.64
Regional background concentration	0.00	-2.55E-02			
Traffic load in 50m	0.41	4.89E-06			
Population in 1000m	0.58	2.88E-04			

Region[a]/determinants	Partial R^2	Beta	$Model_{intra}$[b] R^2/IQR	LAOCV R^2	HV[c] R^2
Squared altitude	0.62	-6.02E-01			
Major road length in 500m	0.64	1.37E-03			
Intercept		2.37E+01			
$PM_{2.5}$ (N^d=119, final model R^2=0.80)			0.48/0.13	0.71	0.71
Regional background concentration	0.68	7.35E-01			
Major road length in 50m	0.79	1.47E-02			
Industry in 5000m	0.80	1.07E-07			
Intercept		4.42E+00			
$PM_{2.5}$ absorbance (N^d=119, final model R^2=0.75)			0.80/0.10	0.68	0.74
Regional background concentration	0.01	6.51E-02			
Traffic load in 50m	0.56	2.78E-07			
Major road length in 1000m	0.69	1.47E-05			
Population in 1000m	0.73	8.33E-06			
Traffic load in major roads in 500m	0.75	2.06E-09			
Intercept		1.03E+00			
**CE**					
NO_2 (N^d=240, final model R^2=0.63)			0.57/0.10	0.36	0.56
Traffic load in 1000m	0.54	5.63E-08			
Traffic intensity to the nearest road	0.60	2.74E-04			
Road length in 50m	0.63	2.02E-02			
Intercept		1.20E+01			
$PM_{2.5}$ (N^d=79, final model R^2=0.82)			0.25/0.48	0.34	0.84
Regional background concentration	0.72	1.17E+00			
Road length in 50m	0.81	8.44E-03			
Traffic load in 100m	0.82	1.76E-07			
Intercept		-2.61E+00			
$PM_{2.5}$ absorbance (N^d=79, final model R^2=0.61)			0.63/0.06	0.55	0.15
Regional background concentration	0.00	8.70E-01			
Traffic load in major roads in 50m	0.38	1.82E-07			

Region[a]/determinants	Partial R^2	Beta	$Model_{intra}$[b] R^2/IQR	LAOCV R^2	HV[c] R^2
Road length in 300m	0.53	1.05E-04			
Natural and green in 5000m	0.61	-1.62E-08			
Intercept	0.00	4.19E-01			
**SE**					
NO_2 (N^d=200, final model R^2=0.75)			0.63/0.25	0.12	0.23
Regional background concentration	0.00	-1.22E+00			
Low residual density in 5000m	0.53	5.42E-07			
Population in 1000m	0.65	1.85E-04			
Traffic intensity to the major road	0.70	3.00E-04			
Road length in 50m	0.75	2.90E-02			
Intercept		1.53E+01			
$PM_{2.5}$ (N^d=80, final model R^2=0.23)			0.50/0.13	0.00	0.00
Road length in 100m	0.10	3.91E-03			
Traffic density in nearest road	0.23	1.56E-04			
Intercept		1.69E+01			
$PM_{2.5}$ absorbance (N^d=80, final model R^2=0.59)			0.67/0.08	0.42	0.16
Regional background concentration	0.01	9.23E-04			
Traffic density in nearest road	0.42	2.15E-05			
Natural in 5000m	0.53	-3.46E-08			
Major road length in 50m	0.59	3.50E-03			
Intercept		2.59E+00			

[a]NE: north Europe; WE: west Europe; CE: central Europe; SE: south Europe. [b]The $Model_{intra}$ R^2s show the median and Inter Quartile Range of the within-area variability explained by the Regional model in individual areas. [c]The HV R^2s represent the correlation between predicted and measured concentrations at validation monitoring sites not used for model building (50% for NO_2, 25% for PM metrics, see methods section). [d]N: number of training sites for modeling.

Table S4. Transferability of the regional models to the independent areas not used for model building [Median(IQR)].

Pollutant/region	Model(R^2)	$TRANS_{intra}$ (R^2) [a]
NO_2		
North	0.67(0.00)	0.71(0.42)
West	0.68(0.00)	0.69(0.16)
Central	0.68(0.00)	0.54(0.25)
South	0.65(0.00)	0.43(0.25)
All[b]	0.68(0.01)	0.58(0.32)
$PM_{2.5}$		
North	0.69(0.04)	0.36(0.35)
West	0.82(0.01)	0.40(0.19)
Central	0.86(0.07)	0.12(0.21)
South	0.71(0.22)	0.31(0.22)
All[b]	0.77(0.17)	0.32(0.28)
$PM_{2.5}$ absorbance		
North	0.69(0.00)	0.55(0.41)
West	0.75(0.00)	0.77(0.30)
Central	0.61(0.00)	0.52(0.19)
South	0.59(0.00)	0.40(0.18)
All[b]	0.69(0.14)	0.49(0.39)

[a]$TRANS_{intra}$: squared correlations between the predictions and observations at independent areas. [b]All: Median and interquartile range of regional model R^2s and $TRANS_{intra}$ R^2s in all the study areas.

Table S5. Model performances of previous large-scale LUR models.

Pollutant and study	Scale	N[a]	Model R^{2b}	HV R^{2c}	RB R^{2d}
**NO_2**					
Hystad et al. (2011)	Canada	134	0.72	—	0.04
Beelen et al. (2009)	Europe	255	0.49	0.39	—
Novotny et al. (2011)	U.S.	423	0.78	0.76	0.15
This study	Europe	960	0.56	0.56	0.08
Vienneau et al. (2013)	Europe	>1500	0.48-0.58	—	0.05
**$PM_{2.5}$**					
Hystad et al. (2011)	Canada	177	0.46	—	0.41
This study	Europe	356	0.86	0.80	0.71
Sampson et al. (2013)	U.S.	903	0.88	—	—
Beckerman et al. (2013)	U.S.	1464	0.63	—	0.52
**Soot**					
Bergen et al. (2013)[e]	U.S.	288	0.79	—	—
This study	Europe	356	0.70	0.70	0.28

[a]N: number of monitoring sites available for model building; all other studies used routine networks and included satellite data as predictors (except Bergen et al.). [b]Model R^2: cross validation R^2 instead of model R^2 for Sampson et al. (2013) and Bergen et al. (2013). [c]HV R^2: Hold out validation. [d]RB R^2: R^2 explained by regional background concentration variable, for all the other studies, the regional background concentration variables was from satellite data. [e]Bergen et al. (2013) reported $PM_{2.5}$ soot as elemental carbon which used thermal measurement method while $PM_{2.5}$ soot in our study was analyzed by optical method.

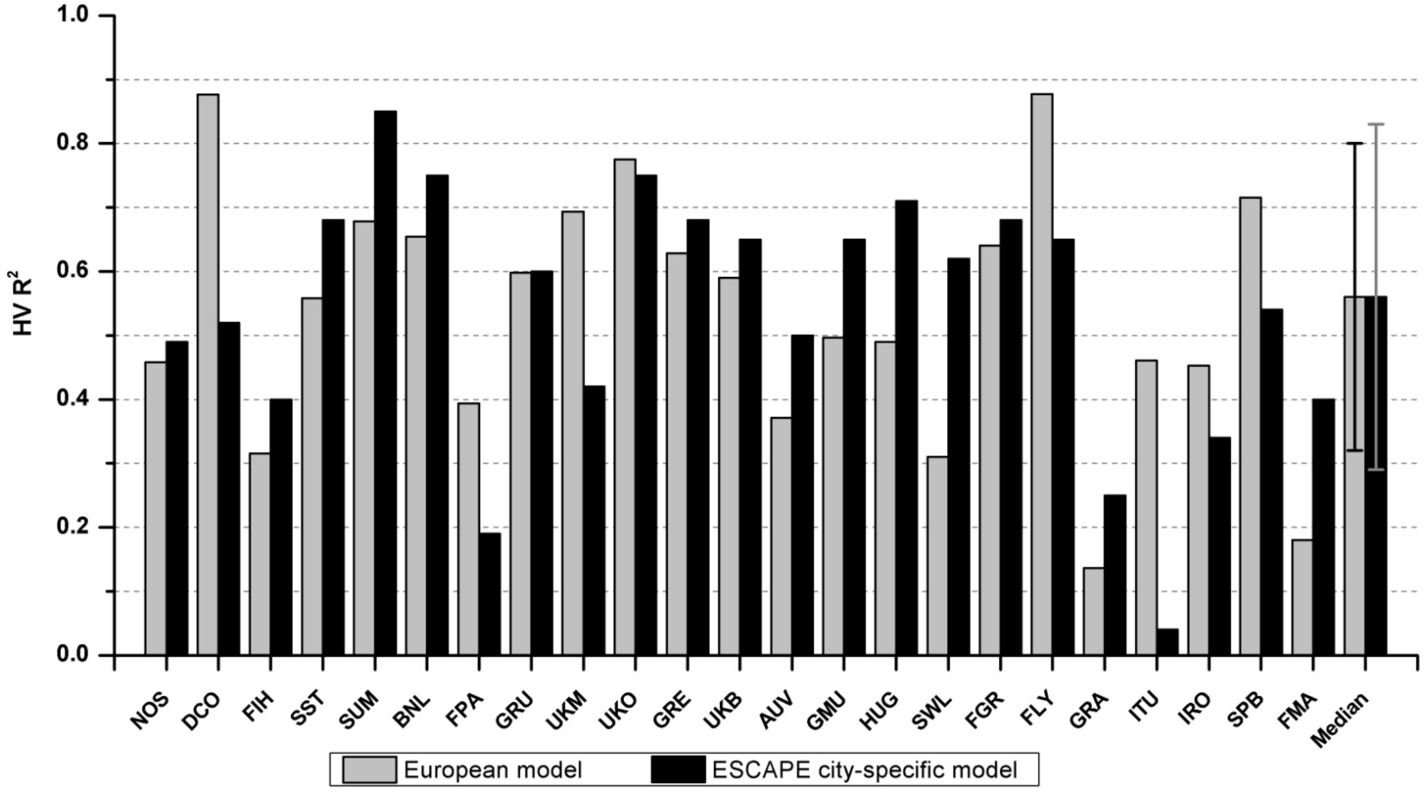

Figure S1. Comparison of HV R^2 between the European model and the ESCAPE city-specific models for NO_2 in 23 study areas as well as median and inter quartile range. Coding of areas please see Table 1.

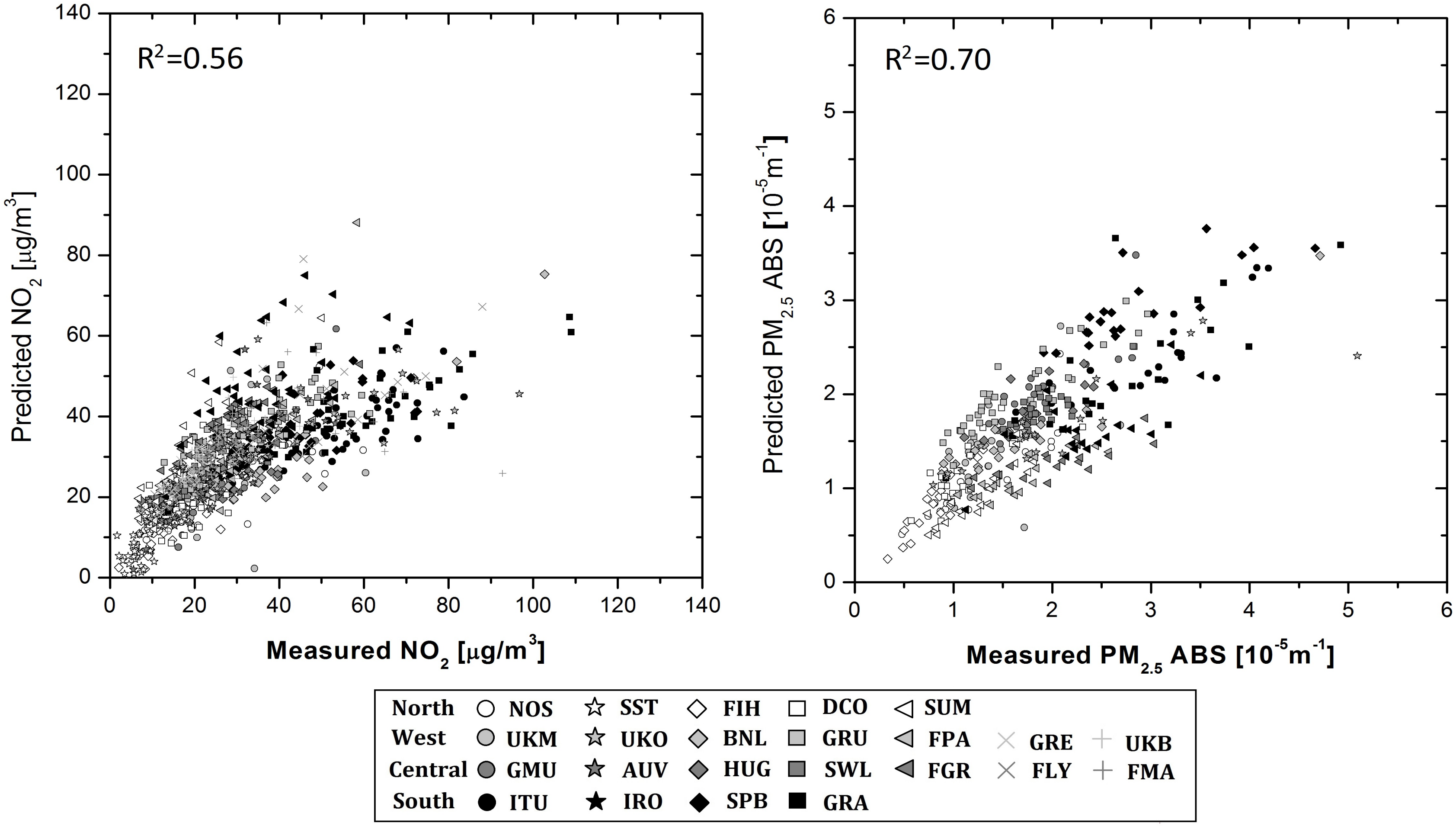

Figure S2. Scatterplot of predicted and measured of NO_2 and $PM_{2.5}$ absorbance with study areas color and symbol coded. Coding of areas please see Table 1.

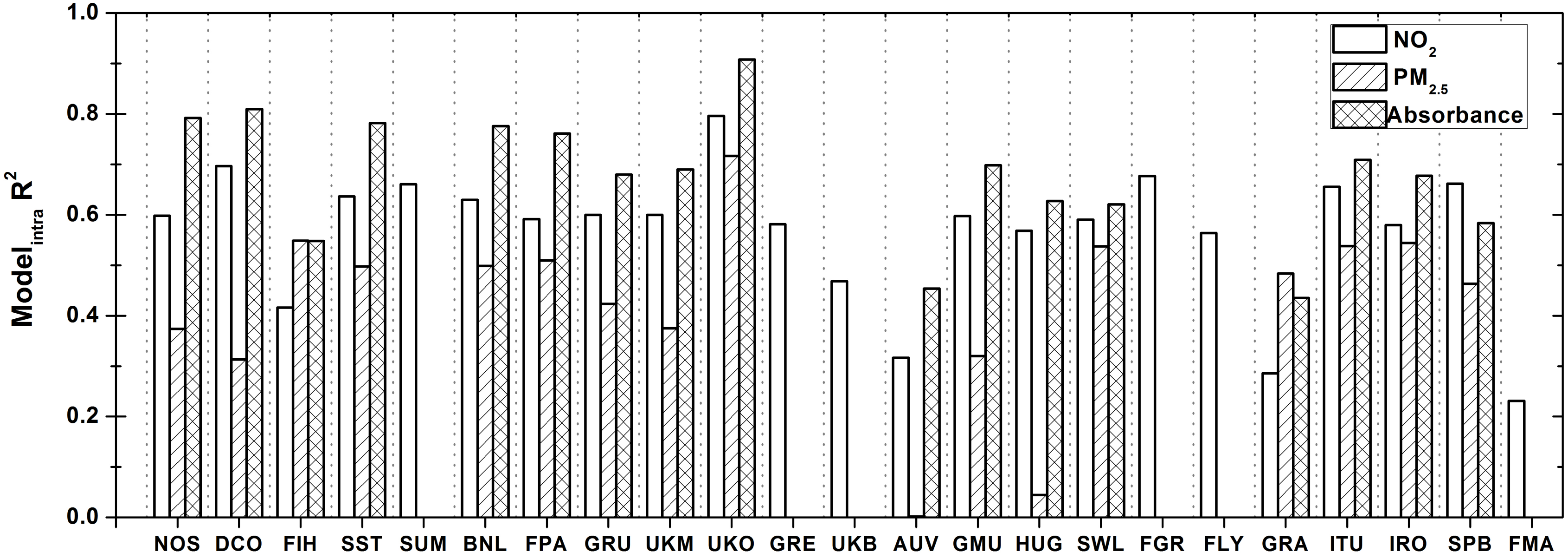

Figure S3. Model$_{intra}$ R^2 of the European models for NO_2 and PM in the 23 study areas. Coding of areas please see Table 1.

References

Beckerman BS, Jerrett M, Serre M, Martin RV, Lee SJ, van Donkelaar A, et al. 2013. A hybrid approach to estimating national scale spatiotemporal variability of PM in the contiguous United States. Environ Sci Technol 47: 7233-7241.

Beelen R, Hoek G, Pebesma E, Vienneau D, de Hoogh K, Briggs DJ. 2009. Mapping of background air pollution at a fine spatial scale across the European Union. Sci Total Environ 407:1852-1867.

Bergen S, Sheppard L, Sampson PD, Kim SY, Richards M, Vedal S, et al. 2013. A national prediction model for pm component exposures and measurement error-corrected health effect inference. Environ Health Perspect 121:1017-1025.

Hystad P, Setton E, Cervantes A, Poplawski K, Deschenes S, Brauer M, et al. 2011. Creating national air pollution models for population exposure assessment in Canada. Environ Health Perspect 119:1123-1129.

Novotny E V, Bechle MJ, Millet DB, Marshall JD. 2011. National satellite-based land-use regression: NO_2 in the United States. Environ Sci Technol 45:4407–4414

Sampson PD, Richards M, Szpiro AA, Bergen S, Sheppard L, Larson TV, Kaufman JD. 2013. A regionalized national universal kriging model using Partial Least Squares regression for estimating annual $PM_{2.5}$ concentrations in epidemiology. Atmos Environ 75: 383-392.

Vienneau D, de Hoogh K, Bechle MJ, Beelen R, van Donkelaar A, Martin R, et al. 2013. Western European land use regression incorporating satellite- and ground-based measurements of NO_2 and PM_{10}. Environ Sci Technol 47: 13555-13564.
